# A Potential Citrate Shunt in Erythrocytes of PKAN Patients Caused by Mutations in Pantothenate Kinase 2

**DOI:** 10.3390/biom12020325

**Published:** 2022-02-18

**Authors:** Maike Werning, Verena Dobretzberger, Martin Brenner, Ernst W. Müllner, Georg Mlynek, Kristina Djinovic-Carugo, David M. Baron, Lena Fragner, Almut T. Bischoff, Boriana Büchner, Thomas Klopstock, Wolfram Weckwerth, Ulrich Salzer

**Affiliations:** 1 Center for Medical Biochemistry, Max Perutz Labs, Medical University of Vienna, 1090 Vienna, Austria; maike.werning@gmail.com (M.W.); v.dobretzberger@gmail.com (V.D.); ernst.muellner@meduniwien.ac.at (E.W.M.); 2 Molecular Systems Biology (MOSYS), University of Vienna, 1010 Vienna, Austria; martin.brenner@univie.ac.at (M.B.); lena.fragner@univie.ac.at (L.F.); wolfram.weckwerth@univie.ac.at (W.W.); 3 Department of Pharmaceutical Sciences/Pharmacognosy, Faculty of Life Sciences, University of Vienna, 1010 Vienna, Austria; 4 Max Perutz Labs, Department of Structural and Computational Biology, University of Vienna, 1010 Vienna, Austria; georg.mlynek@univie.ac.at (G.M.); kristina.djinovic@univie.ac.at (K.D.-C.); 5 Core Facility Biomolecular and Cellular Analysis, University of Natural Resources and Life Sciences, 1190 Vienna, Austria; 6 Department of Biochemistry, Faculty of Chemistry and Chemical Technology, University of Ljubljana, 1000 Ljubljana, Slovenia; 7 Department of Anaesthesia, Intensive Care Medicine and Pain Medicine, Medical University of Vienna, 1090 Vienna, Austria; david.baron@meduniwien.ac.at; 8 Vienna Metabolomics Center (VIME), University of Vienna, 1010 Vienna, Austria; 9 Department of Neurology, Friedrich-Baur-Institute, University Hospital of the Ludwig-Maximilians-University (LMU Klinikum), 80336 Munich, Germany; Almut.Bischoff@med.uni-muenchen.de (A.T.B.); Boriana.Buechner@med.uni-muenchen.de (B.B.); Thomas.Klopstock@med.uni-muenchen.de (T.K.); 10 Munich Cluster for Systems Neurology (SyNergy), 81377 Munich, Germany; 11 German Center for Neurodegenerative Diseases (DZNE), 81377 Munich, Germany

**Keywords:** pantothenate kinase-associated neurodegeneration, neurodegeneration with brain iron accumulation, pantothenate kinase 2, acanthocytes, PANK2 mutations, erythrocyte metabolome

## Abstract

Pantothenate kinase-associated neurodegeneration (PKAN) is a progressive neurodegenerative disease caused by mutations in the pantothenate kinase 2 (PANK2) gene and associated with iron deposition in basal ganglia. Pantothenate kinase isoforms catalyze the first step in coenzyme A (CoA) biosynthesis. Since PANK2 is the only isoform in erythrocytes, these cells are an excellent ex vivo model to study the effect of PANK2 point mutations on expression/stability and activity of the protein as well as on the downstream molecular consequences. PKAN erythrocytes containing the T528M PANK2 mutant had residual enzyme activities but variable PANK2 abundances indicating an impaired regulation of the protein. Patients with G521R/G521R, G521R/G262R, and R264N/L275fs PANK2 mutants had no residual enzyme activity and strongly reduced PANK2 abundance. G521R inactivates the catalytic activity of the enzyme, whereas G262R and the R264N point mutations impair the switch from the inactive to the active conformation of the PANK2 dimer. Metabolites in cytosolic extracts were analyzed by gas chromatography–mass spectrometry and multivariate analytic methods revealing changes in the carboxylate metabolism of erythrocytes from PKAN patients as compared to that of the carrier and healthy control. Assuming low/absent CoA levels in PKAN erythrocytes, changes are consistent with a model of altered citrate channeling where citrate is preferentially converted to α-ketoglutarate and α-hydroxyglutarate instead of being used for de novo acetyl-CoA generation. This finding hints at the importance of carboxylate metabolism in PKAN pathology with potential links to reduced cytoplasmic acetyl-CoA levels in neurons and to aberrant brain iron regulation.

## 1. Introduction

Pantothenate kinase-associated neurodegeneration (PKAN) is a rare hereditary disease (prevalence of 1–2/1,000,000) with progressive neurodegeneration caused by mutations in the pantothenate kinase 2 gene (PANK2) [1,2]. PANK2 is one of three isoforms that mediate the first step in the biosynthesis of coenzyme A (CoA), the carrier of activated acyl chains that is involved in numerous cellular reactions. Classical PKAN is characterized by an early onset (<10 years of age) and rapid progression, whereas atypical PKAN has a later onset and slower disease progression. Patients present with impaired gait, rigidity, impaired balance, or spasticity, and often lose the ability to walk 10–15 years after onset. Episodes of rapid decline are frequently followed by periods of relative stability [2].

MRI evidence for a typical “eye-of-the-tiger” sign in the globus pallidus is a first diagnostic indication to suspect PKAN. The hypo-intensity in this specific region of the brain is due to enhanced iron deposition [2]. PKAN is therefore classified as Neurodegeneration with Brain Iron Accumulation (NBIA) syndrome. Misshaped erythrocytes with thorny protrusions (acanthocytes) occasionally occur in PKAN patients, a finding that is shared with other monogenetic neurodegenerative conditions, such as chorea-acanthocytosis (ChAc) [3] and McLeod syndrome (MLS) [4]. Chorein/VPS13A (ChAc) and the XK protein (MLS) are known peripheral and integral erythrocyte membrane proteins, respectively. 

To detect PANK2 in mature erythrocytes, however, was surprising as it is known to be primarily localized in mitochondria [5,6], which are absent in this cell type. However, our recent study on PKAN erythrocytes revealed that PANK2 actually is the only isoform present and active in erythrocytes [7]. We found that patients who were homozygous or compound heterozygous for the c.1583C > T (T528M) mutation had a significant residual PANK2 activity in their erythrocytes, which correlated with a tendency to slower disease progression. Since erythrocytes—in contrast to CNS neurons—can be easily and abundantly isolated from patient blood samples, they represent an interesting cell model to study the effect of PANK2 mutations on activity and expression/stability of the enzyme with potential insights into the patho-mechanisms of this disease.

The erythrocyte proteome does not only contain the complete set of enzymes required for CoA biosynthesis [8] but actually does synthesize CoA de novo [9], indicating that CoA levels have to be actively maintained throughout the roughly 110-day lifetime of the cell. A lipidomics study on plasma and erythrocyte membrane samples of PKAN patients revealed that PKAN erythrocytes are smaller than cells of control donors and the total phospholipid/cholesterol ratio of the erythrocyte membrane is decreased [10]. PKAN erythrocytes have impaired membrane flexibility, but this altered biophysical property was absent in PKAN blood samples without acanthocytic erythrocytes [11]. Moreover, a cohort of PKAN patients (all homozygous for the c.680 A > G mutation in the PANK2 gene) had variable amounts of acanthocytic cells [12], suggesting that the occurrence of acanthocytosis is influenced by additional factors. While the study by Aoun et al. [10] indicates that a lack of CoA in PKAN erythrocytes causes changes in membrane lipid composition, a thorough elucidation of the associated alterations in cytosolic metabolites has yet to be performed.

In this study, we used a gas chromatography–mass spectrometry-based metabolomics approach to investigate the effect on the erythrocyte metabolome caused by the loss of PANK2 activity. We compared cytosolic metabolite extracts of PKAN patients, related heterozygous carriers, and healthy donors. Furthermore, we assessed residual PANK2 activity and abundance of mutant PANK2 proteins in erythrocytes of patients and carriers and structurally modeled the PANK2 point mutations in order to obtain an insight into the pathological effects of the respective mutations.

## 2. Materials and Methods

### 2.1. Blood Sample Collection

Blood was collected from PKAN patients and related carriers within the Muscle Tissue Culture Collection (Friedrich-Baur-Institute, Klinikum of Ludwig-Maximilians-University of Munich). Nine milliliters of blood was collected into EDTA vacutainers by venipuncture. Informed consent was obtained from each participant (IRB-KUM 45-14). The study was also approved by the ethics committee of the Medical University of Vienna (EK-Nr. 1148/2015). Blood was collected in Munich and sent to Vienna over night at 4–8 °C. Each sampling also included a blood donation from a healthy donor to control for shipment- and time-dependent alterations.

### 2.2. Genetic Evaluation of the Patients

Genetic and clinical data were collected within the Global NBIA Registry (IRB-ID KUM 187-12) and shared for the purposes of this study in a pseudonymized way (Table 1). Within this registry, only the threshold of 6 years of age at the onset of motor symptoms has been chosen as separator between classical and atypical PKAN, without taking into account any other symptoms of the patients or their disease progression. Thus, the “diagnosis” in Table 1 does not reflect the course of the disease.

### 2.3. Pantothenate Kinase (PANK) Activity Assay

PANK2 activity was determined as previously described [7]. Briefly, washed erythrocytes were hypotonically lysed, centrifuged (16,000× *g*, 20 min, 4 °C), and total protein in the supernatant was determined at 280 nm (NanoDrop 2000C, ThermoFisherScientific, Waltham, MA, USA). Then, 250 µg of extract were transferred into a reaction mixture with final concentrations of 11.2 µmol/L ^3^H-D-pantothenic acid (50 Ci/mmol; ART0731; American Radiolabeled Chemicals, St. Louis, MO, USA), 2.5 mmol/L ATP, 10 mmol/L MgCl_2_ and 0.1 mol/L Tris/Cl pH = 7.5 in a total volume of 40 µL and incubated at 37 °C. The reaction mixture was spotted onto an ion-exchange filter (DE81; GE Whatman, Maidstone, UK) and denatured by immersion into 95% ethanol/1% acetic acid. Air-dried filters were transferred into 3 mL scintillation liquid (PerkinElmer, Waltham, MA, USA), and the radioactivity was quantified in a scintillation counter (TRICARB 2100TR, PerkinElmer).

### 2.4. Immunoblot Analysis

Erythrocytes were hypotonically lysed and centrifuged (16,000× *g*, 20 min, 4 °C). For depletion of hemoglobin, the supernatant was passed over a Nickel Sepharose column (GE Healthcare, Chicago, IL, USA), and eluted proteins were concentrated by precipitation with trichloroacetic acid. Samples were resuspended in 0.1% SDS and protein concentration determined via NanoDrop A280. Protein samples were separated by SDS PAGE and transferred to nitrocellulose (GE Healthcare). Membranes were blocked with 5% low-fat dry milk in TBS (150 mM NaCl, 10 mM Tris pH = 7.4), incubated overnight at 4 °C with anti-PANK2 antibody (sc-82288, Santa Cruz Biotechnology, Dallas, TX, USA), and a peroxidase-conjugated goat anti-rabbit IgG, antibody (Jackson ImmunoResearch, West Grove, PA, USA) at room temperature. The chemiluminescence reaction (Pierce ECL Western Blotting Substrate; ThermoFisherScientific) was quantified in a ChemiDoc system (Bio-Rad Laboratories, Hercules, CA, USA). Carbonic anhydrase (CA, rabbit monoclonal anti-carbonic anhydrase 1/CA1 antibody; ab108367, Abcam, Cambridge, UK) was used as a loading control. Optical intensity of the bands was quantified using Image Lab software (Version 6.1, BioRad). 

### 2.5. Gas Chromatography (GC)–Mass Spectrometry (MS)

Erythrocytes were separated from whole blood through centrifugation at 1500× *g* for 10 min at 4 °C and washed three times in phosphate buffered saline (137 mM NaCl, 2.7 mM KCl, 2 mM KH_2_PO_4_, 8 mM Na_2_HPO_4_ × 2H_2_O at pH 7.4). 200 µL aliquots of packed erythrocytes were stored at −150 °C until extraction. For erythrocyte metabolite extraction, 1.05 mL of ice cold extraction solution (methanol:acetonitrile:water, 5:3:2) was added to 150 µL of erythrocytes by vortexing for 30 min at 4 °C. Precipitated proteins were pelleted by centrifugation (21,000× *g*, 15 min, 4 °C) and 950 µL of supernatants was collected, stored at −80 °C for 30 min, and centrifuged again for another 10 min. Five microliters of 10 mM PGP (phenyl-β-D-glucopyranoside) and ten microliters of 2.5 mM raffinose were added as internal standards [13]. Samples were dried under a gentle nitrogen flow and stored at −80 °C until derivatization. Dried samples were derivatized as described [14] by methoximation (20 µL of 40 mg/mL methoxyamine hydrochloride in pyridine, 90 min, 30 °C, followed by silylation (80 µL of N-methyl-N-trimethylsilyl-trifluoroacetamide, 100 g, 30 min, 37 °C). Derivatized samples were transferred into glass vials with inserts and sealed with a crimp cap. Even-numbered n-alkanes (C10–C40) were measured for retention index calculation for each batch.

Derivatized samples were analyzed in various batches on a Pegasus^®^ BT GC-TOF-MS (LECO Corporation, St. Joseph, MI, USA) as described [15]. Each batch consisted of different concentrations of a quality control mix (Table S1), samples, blanks, and an alkane standard for determination of the retention index. Separation of metabolites was achieved by gas chromatography (7890B, Agilent, Santa Clara, CA, USA) on a Rxi-5 ms column (30 m length, 0.25 mm diameter, 0.25 μm film; Restek, Centre County, PA, USA) in split less mode with helium as carrier gas. A fresh Ultra Inlet Liner (78.5 mm length, 900 µL volume; Agilent) was used for each batch. The following settings were applied: flow rate 1 mL/min, injection temperature 230 °C, column temperature start at 70 °C for 1 min, then heating up to 340 °C at a rate of 9 °C/min and a final hold for 15 min. Gas chromatography is coupled to a BT-TOF mass spectrometer (LECO Corporation) with the following settings: ion source temperature 250 °C, acquisition rate 10 spectra/sec, recorded masses 40–600 *m*/*z* with an electron impact (EI) ionization of 70 eV.

For our targeted approach, metabolites were deconvoluted, peak integrated, annotated, and quantified in ChromaTOF (LECO Corporation) based on an in-house reference standard mix quality control. For data processing, first, areas <10,000 were removed from the dataset, then every sample was normalized to the area of the internal standards. 

For untargeted GC-TOF-MS metabolomics, raw measurements were exported as mzML in centroid mode and processed as abf files [16] in MS-Dial (version 4.24; RIKEN) [16,17]. Operations performed were: peak-picking, peak matching, putative metabolite identification (using retention index and FiehnLib [18]), blank filtering, and normalization by sum of the total ion chromatogram (mTIC) [14], respectively. All identified metabolites of interest were checked for accuracy via ChromaTOF (LECO Corporation) and reference libraries NIST (National Institute of Standards and Technology) and GMD (Golm Metabolome Database) as well as an in-house mass spectral library. Metabolites of interest were identified within the two highest out of five levels of confidence levels. Level 1 (L1) is based on reference standard with the exact masses match and retention index of the detected metabolite peaks. Level 2 (L2) is based on library spectrum match and diagnostic evidence as described [19].

Processed data were analyzed by performing multivariate and univariate analyses. Principal component analysis (PCA) and partial least squares-discriminant analysis (PLS-DA) were carried out using MetaboAnalyst [20,21]. The key mass ions representing potential characteristic metabolites were determined based on variable importance in projection (VIP) values obtained from two-way orthogonal comparisons. Mass ions with VIP values greater than one were considered as discriminant key characteristic metabolites. Univariate analyses were performed in parallel to multivariate analyses to identify significant mass ions. One-way Anova with Tukeys HSD post hoc test and false discovery rate (FDR, cut-off ≤ 0.05) correction was performed using MetaboAnalyst to determine the significantly changed mass ions between control, carrier, and patient groups. MetaboAnalayst and the Kyoto Encyclopedia of Genes and Genomes (KEGG) database were used to analyze and visualize the affected pathways. 

### 2.6. Molecular Visualization

PANK proteins are highly cooperative allosteric enzymes that exploit structural changes at the dimer interface to coordinately switch between active and inactive conformations [22]. To illustrate this conformational change, PANK3 with acetyl-CoA (PDB 3mk6) representing the inactive “open” conformation and PANK2 structure in complex with pantothenate and adenosine diphosphate (PDB 5e26) representing the active “closed” conformation were taken. The catalytic core of the different PANK isoforms is highly conserved indicating a common catalytic mechanism of all isoforms. Figures of protein structures were created using pymol (https://pymol.org/2/, accessed on 25 November 2021).

### 2.7. Statistical Analysis

Univariate analysis and Tukey boxplots were performed in Prism 6 (GraphPad, San Diego, CA, USA). Multivariate data analysis was performed in MetaboAnalyst. All other statistical analyses were performed as reported in the section gas chromatography (GC)–mass spectrometry (MS) above. 

## 3. Results

### 3.1. Residual Activity and Abundance of PANK2 Mutant Proteins

Our recent previous study on PKAN erythrocytes revealed that 50% of the patients in our cohort exhibited residual PANK2 activity in their erythrocytes [7]. In the present study, erythrocytes of patients P1–P3 had PANK2 activities of 12–17% as compared to those of healthy controls, whereas no PANK2 activity was found in cells of patients P4–P6 (Table 2). Patients P1 and P2 were already included in our previous study. Their re-sampled erythrocytes showed similar activities to those already determined earlier, suggesting that residual PANK2 activity is a constant characteristic of the respective PANK2 mutants. All three patients with residual activity harbor the c.1583C > T, p. T528M mutation on one allele (Table 1). Whereas P3 has a deletion mutation on its second PANK2 allele, P1 and P2 harbor the c.1561G > A, p. G521R, which results in the catalytically inactive point mutant PANK2 G521R.

**Figure 1:**
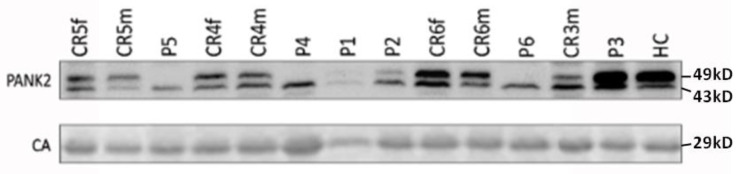
Abundance of PANK2 protein in erythrocytes of patients affected by PKAN and disease carriers. Hemoglobin-depleted cytosolic extracts were resolved by SDS PAGE and analyzed by Western blotting on a nitrocellulose membrane using a polyclonal rabbit anti-PANK2 antibody. Carbonic anhydrase (CA) was detected by a rabbit monoclonal antibody and served as loading control to assess the specific abundance of the PANK2 protein. HC refers to a sample obtained from a healthy control donor used for normalization to calculate the relative abundance of PANK2 in the patient and carrier samples.

**Figure 2:**
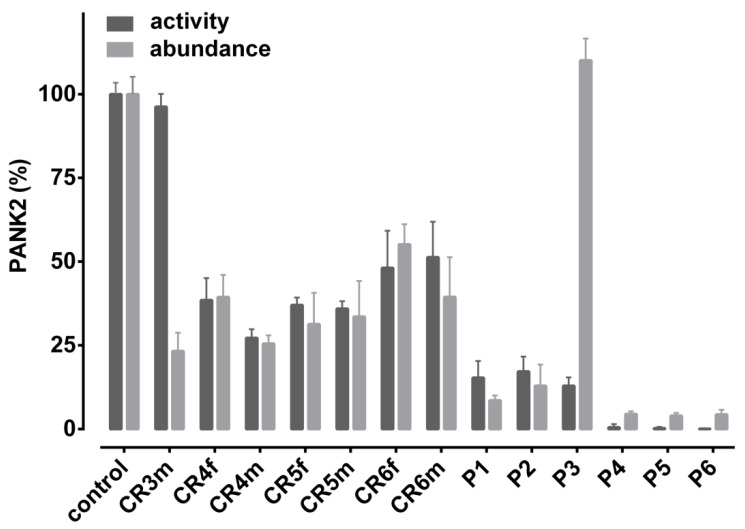
Comparison of PANK2 activity and abundance in erythrocytes of patients affected by PKAN and disease carriers. PANK2 activity and abundance as assessed by a radiometric assay and Western blotting, respectively, are given in percent normalized to that of a healthy control donor, which is set to 100%. Data are derived from Table 2 showing mean values with standard deviations.

To estimate the contribution of reduced protein expression/stability on the overall loss in enzymatic activity, we performed Western blot analyses of hemoglobin-depleted extracts from erythrocyte cytosolic fractions. The Western blot (Figure 1) reveals a major band at 49 kDa, which conforms to the reported size of PANK2 in other cell types [7], and a minor band at about 43 kDa, which is likely due to a cross-reaction of the antibody. The band of the major erythrocyte cytosolic protein carbonic anhydrase (CA) served as loading control. The 49 kDa PANK2 band is markedly reduced in patients P4–P6, reduced in patients P1 and P2, and—surprisingly—very abundant in patient P3 (Figure 1). The respective values are given as means of three independent sample runs in Table 2. Together, the activity and expression data of patient P4 being homozygous for the PANK2 G521R mutant suggest that this point mutation impairs both the expression/stability and activity of the PANK2 protein. In contrast, the PANK2 T528M point mutant (patient P3) is highly stable, but the protein seems to have a low specific activity. Co-expression of PANK2 G521R and PANK2 T528M point mutants (patients P1 and P2) results in strongly reduced overall abundance of PANK2 mutant protein. The specific activity of the residual PANK2 protein, however, is apparently similar or even higher than that of WT PANK2 (as can be assessed by the ratio of residual activity over residual abundance in Table 2 and Figure 2).

**Figure 3:**
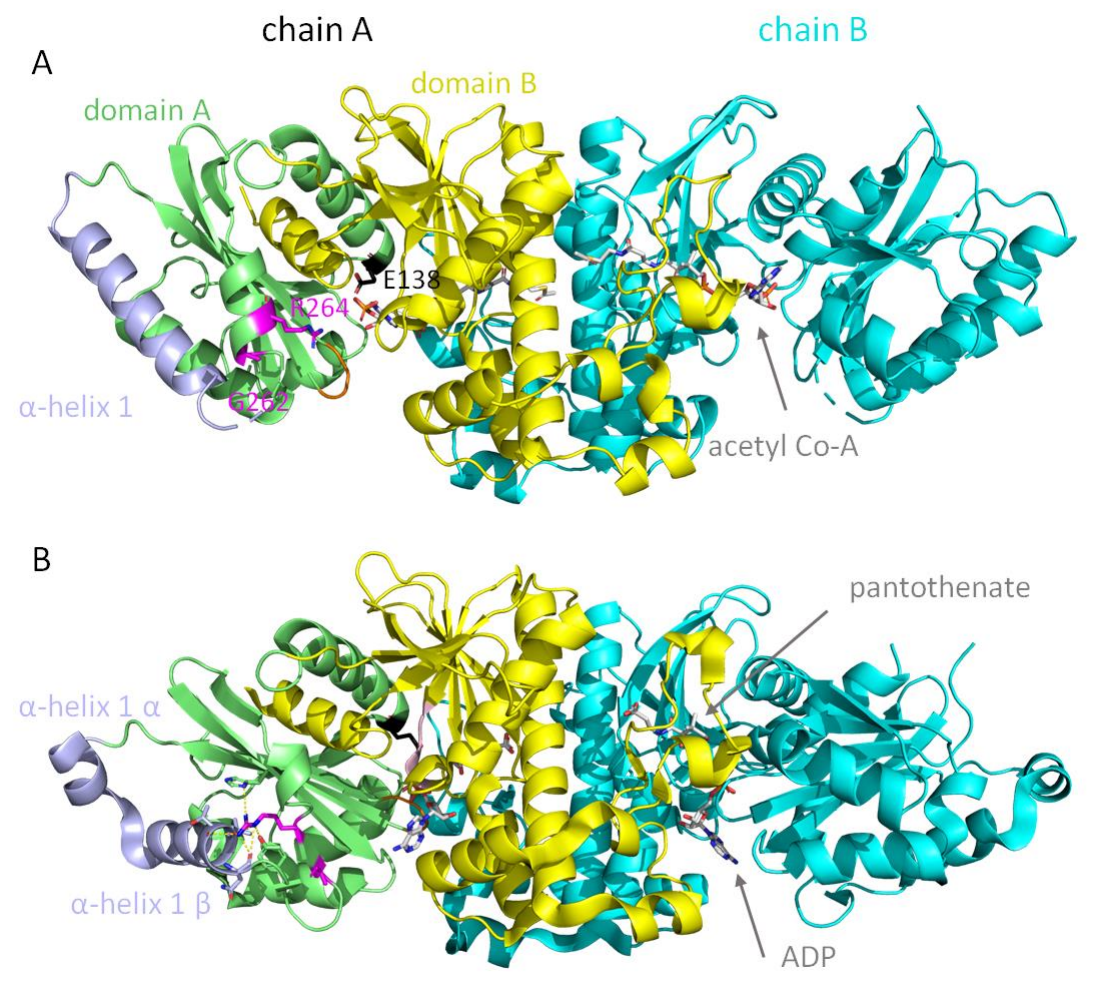
Conformational changes between inactive and active states of PANK and movement of G262 and R264. (**A**) PANK3 dimer structure (PDB 3mk6) (inactive conformation) is shown in cartoon representation. Domains A and B of the first subunit are shown in lime and yellow, respectively, and the chain B in cyan. Acetyl-CoA is shown as sticks. The glycine-rich ATP binding loop is highlighted in orange and the short ATP binding helix in light pink. Alpha-helix 1 is shown in light blue and G262 and R264 are shown in magenta as sticks. Catalytic residue E138 is shown as black sticks. (**B**) PANK2 dimer structure (PDB 5e26) (active conformation) is shown in cartoon representation. Upon release of acetyl-CoA a conformational switch happens in domain A. Alpha-helix 1 is broken into helix1α and helix1β. R264 is rotated 180° and makes many polar and ionic interactions with residues of domain A. ADP and pantothenate are shown as sticks. Yellow dashes indicate ionic and hydrogen bonding of R264 to several amino acids of domain A.

Patients P5 and P6 are compound heterozygous for the G521R/G262R mutations and the R264N/L275fs mutations, respectively. Both patients reveal very low expression/stability of PANK2 protein and no residual PANK activity in their erythrocytes. The two point mutations G262R and R264N are both localized in a loop between alpha-helix 1 and 2 of domain A of the protein (Figure 3). Molecular modeling reveals that this region undergoes drastic conformational changes in the transition between the active and the inactive state of the enzyme. Thus, these mutations likely affect the stability of the protein and/or lock it in the inactive conformation.

### 3.2. Metabolomic Analysis of PKAN Blood Samples

**Figure 4:**
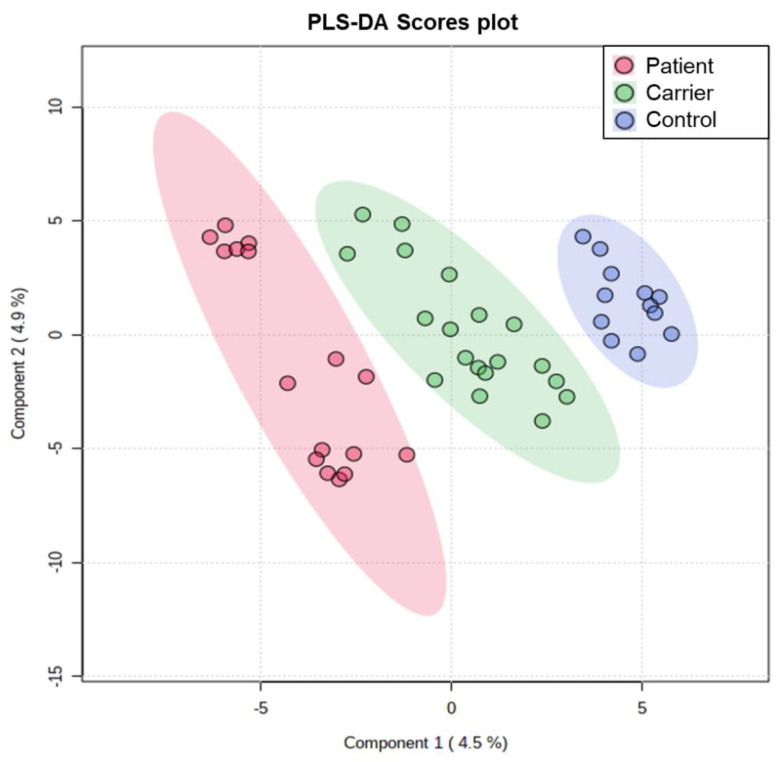
Partial least square-discriminant analysis (PLS-DA). Sample distributions around Principal Components (PC) 1 and 2 are shown. PC1 and PC2 explain 4.5% and 4.9% of the variance, respectively. Despite the recognized influence of biologic variability on the erythrocyte metabolome partial least square discriminant analysis shows that metabolic variance is explained by PKAN. The upper, slightly separate group of data is the sample set of the twin brothers with the T528M/G521R PANK2 mutations.

We further used metabolomics to address the consequences of a (nearly) complete loss of PANK2 activity in erythrocytes of PKAN patients (Table S2). Analyzing metabolite levels in erythrocyte cytosolic extracts revealed significant alterations in PKAN erythrocytes as compared to that of carrier and healthy control (Table 3). Despite the known donor- and storage-dependent metabolic variability in erythrocyte samples [23], the FDR of less than 0.05 and the PLS-DA (Figure 4) both indicate that the observed changes in key metabolites are significant and caused by the PKAN condition.

**Figure 5:**
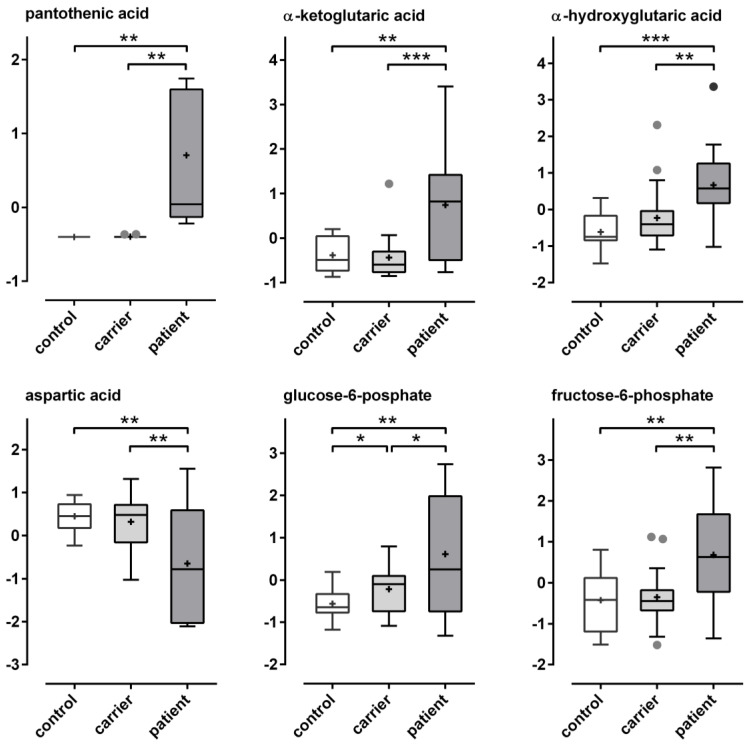
Boxplots of selected metabolites reveal significant alterations in PKAN erythrocytes. The z-normalized data of selected metabolites grouped in patient, carrier, and control samples are given in box-and-whisker plots (GraphPad Prism). Grey points indicate outliers and black crosses mean values. Differences between the groups were analyzed by Student’s *t*-tests and significant results are indicated by asterisks (* = *p* ≤ 0.05, ** = *p* ≤ 0.01, *** = *p* ≤ 0.001). Boxplots of the other metabolites from Table 3 are given in Figure S1.

Pantothenate levels of red blood cell cytosol were elevated in patients relative to carrier and controls. This is in line with the interpretation that cytosolic pantothenate is readily consumed by the CoA biosynthetic pathway under normal physiological conditions but accumulates in PKAN erythrocytes. Moreover, as a consequence, this implicates a lack of CoA and its many acyl derivates in patient derived red blood cells. Our GC-based metabolomics approach did not allow for the direct assessment of CoA metabolites. However, we found significantly elevated levels of the carboxylic acids α-ketoglutarate and α-hydroxyglutarate as well as reduced levels of aspartate in the erythrocyte cytosol of PKAN patients (Figure 5 and Table 3). Furthermore, PKAN erythrocytes had significantly increased amounts of the first two metabolites of the glycolysis pathway, glucose-6-phosphate, and fructose-6-phosphate (Figure 5 and Table 3). 

**Figure 6:**
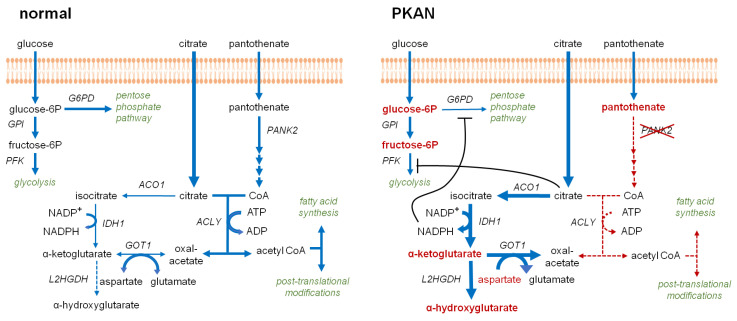
Pathway model of a potential citrate shunt in PKAN erythrocytes. In erythrocytes, citrate imported from the plasma is the only source for the acetyl moiety in acetyl-CoA, which is the primary building block for fatty acid synthesis and essential for post-translational modifications. Its formation is mediated by ACLY under consumption of ATP and citrate. Depletion of CoA largely impairs this reaction in PKAN erythrocytes. An alternative citrate pathway via ACO1 and IDH1 is preferably active in PKAN erythrocytes since metabolites of this route were found to be altered accordingly. Metabolites with normal abundances are in black, pathways are in italic green font. Altered metabolites are shown in red, normal font indicates decreased and bold fonts increased levels. Pathways that are strongly affected in PKAN erythrocytes are indicated with dotted lines and arrow heads in red. The red cross indicates absent/inactive PANK2 protein due to the genetic condition. Black arcs indicate potential inhibition of G6PD by elevated NADPH/NADP^+^ ratios and of PFK by increased citrate and ATP levels. The following enzyme acronyms are used: aconitate hydratase (ACO1), isocitrate dehydrogenase (IDH1), aspartate-aminotransferase (GOT1), ATP-citrate synthase (ACLY), α-hydroxyglutarate dehydrogenase (L2HGDH), glucose-6-phosphate isomerase (GPI), glucose-6-phosphate dehydrogenase (G6PD), phosphofructokinase (PFK), and pantothenate kinase 2 (PANK2).

Since erythrocytes lack acetyl-CoA synthetase (ACSS2) [8,24] the CoA-dependent breakdown of citrate by ATP-citrate synthase (ACLY) is the only source for acetyl-CoA, which in turn is crucial for many cellular reactions, e.g., lipid biosynthesis and protein acetylation. Lack of CoA in PKAN erythrocytes likely impairs this route of the citrate metabolism. In fact, a potential alternative citrate utilization pathway (citrate shunt) fits well to the metabolic alterations found in PKAN erythrocytes. A scheme integrating these observations into a coherent pathway model of metabolic shifts in PKAN erythrocytes is presented in Figure 6 and discussed below.

## 4. Discussion

This study reveals altered carboxylate metabolism in erythrocytes of patients suffering from pantothenate kinase-associated neurodegeneration (PKAN) and describes the molecular effects of PANK2 point mutants on expression/stability and activity of the enzyme.

In erythrocytes, CoA is involved in the following metabolic pathways: (i) as a long-chain acyl carrier in phospholipid turnover and reversible palmitoylation of membrane proteins; (ii) as a short- and intermediate-chain length acyl carrier in long-chain fatty acid synthesis; and (iii) as a carrier for activated acetyl groups required for fatty acid synthesis and posttranslational protein modification. Since erythrocytes lack pyruvate dehydrogenase complex-mediated and ACSS2-mediated [8,24] acetyl-CoA generation, ATP and CoA-dependent breakdown of citrate via ACLY, an abundant enzyme in the erythrocyte cytosol, is the only way for acetyl-CoA production in these cells. Recently, quantitative tracing metabolic experiments revealed that citrate consumption significantly contributes to the metabolism of erythrocytes [25]. Despite the lack of CoA, citrate levels in PKAN erythrocytes were not significantly elevated (data not shown), suggesting a(n) altered citrate usage pathway(s) in these cells. In line, we find significantly increased levels of α-ketoglutarate (Figure 5), indicating that citrate is converted to isocitrate by aconitase (ACO1) and subsequently decarboxylated by isocitrate dehydrogenase (IDH1). The latter reaction is coupled to NADPH production. We further observed significantly elevated versus reduced levels of α-hydroxyglutarate and aspartate (Figure 5), respectively, indicating that α-ketoglutarate is reduced to α-hydroxyglutarate and transaminated by the aminotransferase GOT1 to glutamate under consumption of aspartate. The increase in glucose-6-phosphate and fructose-6-phosphate in PKAN erythrocytes is likely due to feedback inhibitions of both the glycolysis and the pentose phosphate pathway (PPP). In consequence, (i) glucose-6-phosphate dehydrogenase (G6PD), catalyzing the first and rate limiting step of the PPP, is inhibited by high NADP/NADP^+^ ratios due to the increased IDH1-mediated α-ketoglutarate generation in PKAN erythrocytes. (ii) Phosphofructokinase (PFK) controls the progression into the glycolytic pathway and is allosterically inhibited by higher levels of ATP. This inhibition would then be synergistically enhanced in the presence of elevated citrate concentrations [26]. Since ACLY-mediated acetyl-CoA generation consumes both ATP and citrate, loss of this reaction and the concomitant reduction in acetyl-CoA-dependent biosynthetic pathways likely reduce the glycolytic flux in PKAN erythrocytes, resulting in the accumulation of glucose-6-phosphate and fructose-6-phosphate. The model in Figure 6 summarizes these considerations and names the enzymes that are suggested to be involved and known to be abundant in erythrocytes [8].

The limitations of this study are (i) the relatively low number of different patients and (ii) the fact that intracellular concentrations of CoA and its various acyl-derivatives could not be assessed. Thus, the notion of reduced/absent CoA levels in PKAN erythrocytes (as depicted in the model of Figure 6) is based on the assumption that de novo biosynthesis involving PANK2-mediated pantothenate phosphorylation as its first step is the only way for cellular CoA. However, recent data somehow questioned this view and suggested that membrane passage of extracellular CoA intermediates such as 4′-phosphopantetheine might represent an alternative route [27,28]. Moreover, a study employing a PKAN mouse model revealed that dietary uptake of 4′-phosphopantetheine could rescue changes in CoA-, iron-, and dopamine-related biomarkers as well as activities of mitochondrial enzymes [29]. These findings on the one hand open a thrilling perspective on a novel treatment option for PKAN patients, and, on the other hand, point to as yet to be explored mechanisms of intra- and intercellular transport of CoA intermediates. With respect to erythrocytes, 4′-phosphopantetheine in the blood plasma would have to pass the plasma membrane in order to rescue CoA levels in PKAN erythrocytes. Neither physiologic levels of 4′-phosphopantetheine in human plasma nor respective uptake mechanisms by erythrocytes are known to date, yet it cannot be excluded that such an alternate CoA replenishment route is actually present in human erythrocytes. However, in view of the various alterations in PKAN erythrocytes described in this and previous studies [7,10,11,12], it is very unlikely that such a non-canonical pathway could reconstitute erythrocyte CoA levels to their full extent. Thus, even if loss of CoA is partially rescued, the (reduced) CoA level will likely affect the activity of ACLY, since it is the most abundant CoA-binding enzyme in erythrocytes [8]. Though not directly assessed in this study, enhanced citrate availability due to CoA-limited ACLY activity is a plausible explanation for the metabolic alterations in PKAN erythrocytes (Figure 6). 

Given the central role of ACLY-mediated acetyl-CoA generation in the cellular metabolism, we might extend the view from the quite simple situation in erythrocytes and envision to the more complex situation in neurons and most other cells where mitochondrial metabolism and other PANK isoforms have to be considered. In these cells, acetyl-CoA is mainly produced from pyruvate and fatty acids within mitochondria, where it is consumed in the citrate generating step of the TCA cycle. Acetyl-CoA cannot directly be exported to the cytosol but is shuttled as citrate via the tricarboxylate transporter SLC25A1 [30] to fuel cytoplasmic ACLY-mediated acetyl-CoA generation [31]. While PANK isoforms are located at different subcellular sites, it is still not clear where the further steps of CoA biosynthesis take place and how CoA is distributed among the different subcellular organelles and the cytoplasm [32]. However, largely reduced energy production and severe mitochondrial dysfunction in neurons and peripheral nerves were found in PANK2 knockout mice [33], indicating that CoA deficiency directly affects mitochondria. Thus, it can be hypothesized that mitochondrial citrate production and export is considerably reduced in PKAN neurons and that cytoplasmic citrate levels and acetyl-CoA production become more dependent on extracellular citrate. Further, in contrast to the situation in erythrocytes, in neurons of PKAN patients ACLY activity might be limited by cytoplasmic citrate rather than cytoplasmic CoA. Citrate-limited ACLY activity might be specifically detrimental for cholinergic neurons that highly express this enzyme in their synapses for acetylcholine synthesis. Finally, since cytoplasmic citrate levels are linked to the expression of hepcidin [34,35], altered citrate metabolism, hypothetically, could be associated with aberrant iron regulation and brain iron accumulation in PKAN. These considerations open the perspective on potential treatment options for PKAN patients based on targeted supplementation. CoA intermediates as well as metabolites of the inter- and intra-cellular citrate pathways should be considered. However, to this end, we have to learn much more about metabolic fluxes at the organismal level and their changes during development. PKAN animal models may therefore be useful to study the metabolic aspect of this disease and help to identify supplements that potentially can slow down the progression of neurodegeneration. Given the links between PKAN and Parkinson’s disease, such studies would also be relevant for more common neurodegenerative diseases.

Erythrocytes are an interesting cell model to study molecular aberrations in PKAN, since PANK2 is the only PANK isoform present in these cells [7]. This is a particularly valuable circumstance when exploring the effect of point mutations in general as well as the interference of particular point mutations in case of a compound heterozygous genotype. The PANK2 T528M mutation is of special interest as it is the most common of all point mutations associated with PKAN pathology. Studies on recombinant PANK2 T528M mutants revealed normal (even slightly elevated) catalytic activity as compared to WT PANK2 [22,36]. In line, molecular modeling of this mutant did not infer any structural or functional impact of this mutation. Erythrocytes of patients homozygous for the T528M mutation had considerable residual PANK2 activities of 40% and 56% [7]. In this study, patient P3 having the T528M mutation combined with a deletion mutation on the other allele had a residual erythrocyte PANK2 activity of 12% (Table 2). WB analysis, however, revealed high amounts of PANK2 protein in erythrocytes (Figure 1 and Table 2), suggesting that inactive PANK2 T528M mutant protein massively accumulates in these cells without being removed by proteolysis. As previously outlined [7], the T528M PANK2 mutation likely abrogates a phosphorylation site in the C-terminal region, which could be essential for the regulation of the catalytic activity and/or proteasomal degradation of misfolded protein. Patients P1 and P2 harbor a combination of the T528M mutation and the inactivating G521R mutation. Their erythrocytes exhibit markedly reduced PANK2 abundance, but similar or even higher residual activity than the erythrocytes of patient P3. Possibly, mixed dimers of these two PANK2 mutants are active since the T528M mutant monomer contributes its catalytic activity but does not aggregate while the G521R mutant monomer is still capable of posttranslational regulation via the T528 site.

The deleterious effects of mutants G262R and R264N (Patients 5 and 6, respectively) can be explained on the atomic level by analyzing the crystal structures of PANK3 with acetyl-CoA (inactive conformation) and PANK2 with pantothenate and adenosine diphosphate (active conformation) bound (Figure 3). PANK proteins are highly cooperative allosteric enzymes. The switch from the inactive to the active state is characterized by large conformational rearrangements [22,37]. Residues G262 and R264 are situated in a loop between alpha-helix 1 and 2 [37]. When the feedback inhibitor acetyl-CoA is bound the kinase is in the “open” inactive conformation. Upon release of acetyl-CoA and subsequent binding of ATP, domain A undergoes a massive structural rearrangement, whereby helix 1 breaks into two halves, helix1α and helix1β, and the arginine rotates 180 degrees inwards. Biochemical studies on recombinantly expressed PANK3 [22] and PANK2 mutants [36] are in line with our consideration that a mutation at position 264 either affects the stability of the active conformation or impairs the switch from the inactive to the active conformation. 

## 5. Conclusions

PKAN is a relentlessly progressive neurodegenerative disease caused by mutations in the PANK2 gene that result in insufficient CoA production. Although molecular patho-mechanisms are still far from being resolved, it is conceivable that CoA levels fall below a critical concentration in specifically vulnerable cells in the brain causing a fatal metabolic imbalance. Since CoA is a cofactor in a myriad of cellular reactions, it is essential to identify the pathways and cell types that are most affected. This study reveals that PKAN erythrocytes have an altered carboxylate metabolism likely due to CoA-limited ACLY activity, and thus have enhanced citrate availability. ACLY uses citrate to generate acetyl-CoA, the essential building block for lipid biogenesis and the substrate for ubiquitous posttranslational modifications. While erythrocytes seem to be able to cope with reduced acetyl-CoA levels, neurons and other brain cells are critically affected. It can be hypothesized that altered intermediary citrate metabolism is part of this fatal cellular dysregulation in these cells. Recent studies on PKAN animal models raise the perspective that dietary supplementation with CoA (intermediates) could be a treatment option for PKAN patients. Our study, in addition, suggests that basic metabolic pathways should also be considered as potential targets for guided supplementation. We still have to learn much more about metabolic fluxes at an organismal level; however, an increase in respective knowledge will be the basis for identifying supplements that might compensate critical metabolite losses in PKAN patients, and thereby eventually slow down or even prevent progression of the disease. 

## Figures and Tables

**Table 1:** Clinical and genetic data of PKAN patients. The DNA mutations refer to the NCBI Reference Sequence: NM_153638.3 (pantothenate kinase 2 mRNA transcript variant 1). In the registry “diagnosis”, the threshold of 6 years of age at the onset of motor symptoms was chosen as only discriminant between “classical” and “atypical” and does not reflect the course of the disease.

Patient	Gender	Age of Onset	Diagnosis	DNA Mutation		Protein Mutation	
				Allele 1	Allele 2	Allele 1	Allele 2
P1	♂	12	atypical	c.1561G > A	c.1583C > T	G521R	T528M
P2	♂	13	atypical	c.1561G > A	c.1583C > T	G521R	T528M
P3	♀	2	classical	c.1583C > T	deletion exon 2–4	T528M	del
P4	♀	3	classical	c.1561G > A	c.1561G > A	G521R	G521R
P5	♂	1	classical	c.1561G > A	c.784G > C	G521R	G262R
P6	♂	4	classical	c.791G > A	c.823_824del	R264N	L275fs

**Table 2:** PANK2 activities and abundances in erythrocyte of PKAN patients and carriers. Erythrocyte cytosolic extracts of a healthy donor (control), carriers (CR3-CR6 (f/m denotes father or mother of respective patient)), and PKAN patients (P1–P6) were assayed by radiometry for phospho-pantothenate generation described in Section 2. Specific PANK2 activities per total protein were determined and relative PANK2 activities were calculated from the specific activities by normalization to the specific activity of the control donor. Erythrocyte cytosolic extracts of the same set of blood samples were depleted of hemoglobin concentrated and analyzed by SDS PAGE and Western blotting for the abundance of PANK2 protein as described in Section 2. Specific PANK2 abundances were determined in relation to carbonic anhydrase and relative PANK2 abundances were again calculated by normalization to that of the control donor. All values are mean values (*n* = 3) given in relative percent plus/minus standard deviations (stdev.).

	Activity (% ± stdev.)	Abundance (% ± stdev.)
control	100.00 ± 0.00	100.00 ± 0.00
CR3m	96.25 ± 3.82	23.28 ± 5.46
CR4f	38.50 ± 6.55	39.42 ± 6.60
CR4m	27.21 ± 2.59	25.53 ± 2.44
CR5f	37.03 ± 2.24	31.32 ± 9.37
CR5m	35.93 ± 2.24	33.58 ± 10.6
CR6f	48.14 ± 11.1	55.12 ± 6.03
CR6m	51.29 ± 10.6	39.46 ± 11.8
P1	15.35 ± 4.94	8.55 ± 1.49
P2	17.19 ± 4.43	12.89 ± 6.38
P3	12.91 ± 2.50	110.10 ± 6.52
P4	0.54 ± 0.94	4.5 ± 0.81
P5	0.24 ± 0.42	3.98 ± 0.86
P6	0.12 ± 0.12	4.35 ± 1.41

**Table 3:** Cytosolic metabolites significantly altered in erythrocytes of PKAN patients. List of targeted (^#^) and untargeted cytosolic metabolites with significant alterations in PKAN erythrocytes as compared to that of carrier and healthy donors. Normalized to the internal standard (targeted metabolites), or to mTIC (untargeted metabolites), one-way Anova with a Tukey’s HSD post hoc test and false discovery rate (FDR, cut-off ≤ 0.05) correction was performed using MetaboAnalyst as described in Section 2; ID confidence represents the level of metabolite identification (highest L1 to lowest L5) as described in Section 2. Asterisks denote significance between groups at *p*-Value (<0.05 (*), <0.01 (**), <0.001 (***), <0.0001 (****)).

Metabolites	FDR	Significance	Tukey’s HSD	*p*-Value	ID-Confidence
^#^ pantothenic acid	0.000117	****	patient-carrier; patient-control	3.76 × 10^−7^	L1
quinic acid	0.000731	****	control-carrier; patient-carrier	4.7 × 10^−6^	L2
2-hydroxyvaleric acid	0.001771	****	patient-carrier; patient-control	2.33 × 10^−5^	L2
^#^ α-ketoglutaric acid	0.006476	***	patient-carrier; patient-control	0.000125	L1
fructose	0.01230	***	patient-carrier; patient-control	0.000277	L2
^#^ α-hydroxyglutaric acid	0.01670	***	patient-carrier; patient-control	0.000437	L2
fructose-6-phosphate	0.02248	***	patient-carrier; patient-control	0.000745	L2
asparagine	0.02248	***	control-carrier; patient-control	0.000795	L2
^#^ aspartic acid	0.03486	**	patient-carrier; patient-control	0.001395	L1
glycerol-3-galactoside	0.03486	**	patient-carrier	0.001457	L2
glucose-6-phosphate	0.04188	**	patient-carrier; patient-control	0.001885	L2
mannonic acid	0.04470	**	patient-carrier; patient-control	0.002156	L2

## Data Availability

All data of this manuscript are available upon request.

## Supplementary Materials

The following are available online at https://www.mdpi.com/article/10.3390/biom12020325/s1, Figure S1: Boxplots of further metabolites altered PKAN erythrocytes, Table S1: Linear range of QCs and samples, Table S2: Normalized GC-MS data from targeted and untargeted metabolites.
