# Supplementary figures and images for: A Potential Citrate Shunt in Erythrocytes of PKAN Patients Caused by Mutations in Pantothenate Kinase 2
Source: Biomolecules. 2022 Feb 18;12(2):325. doi: 10.3390/biom12020325 (PMC8869601; doi:10.3390/biom12020325)

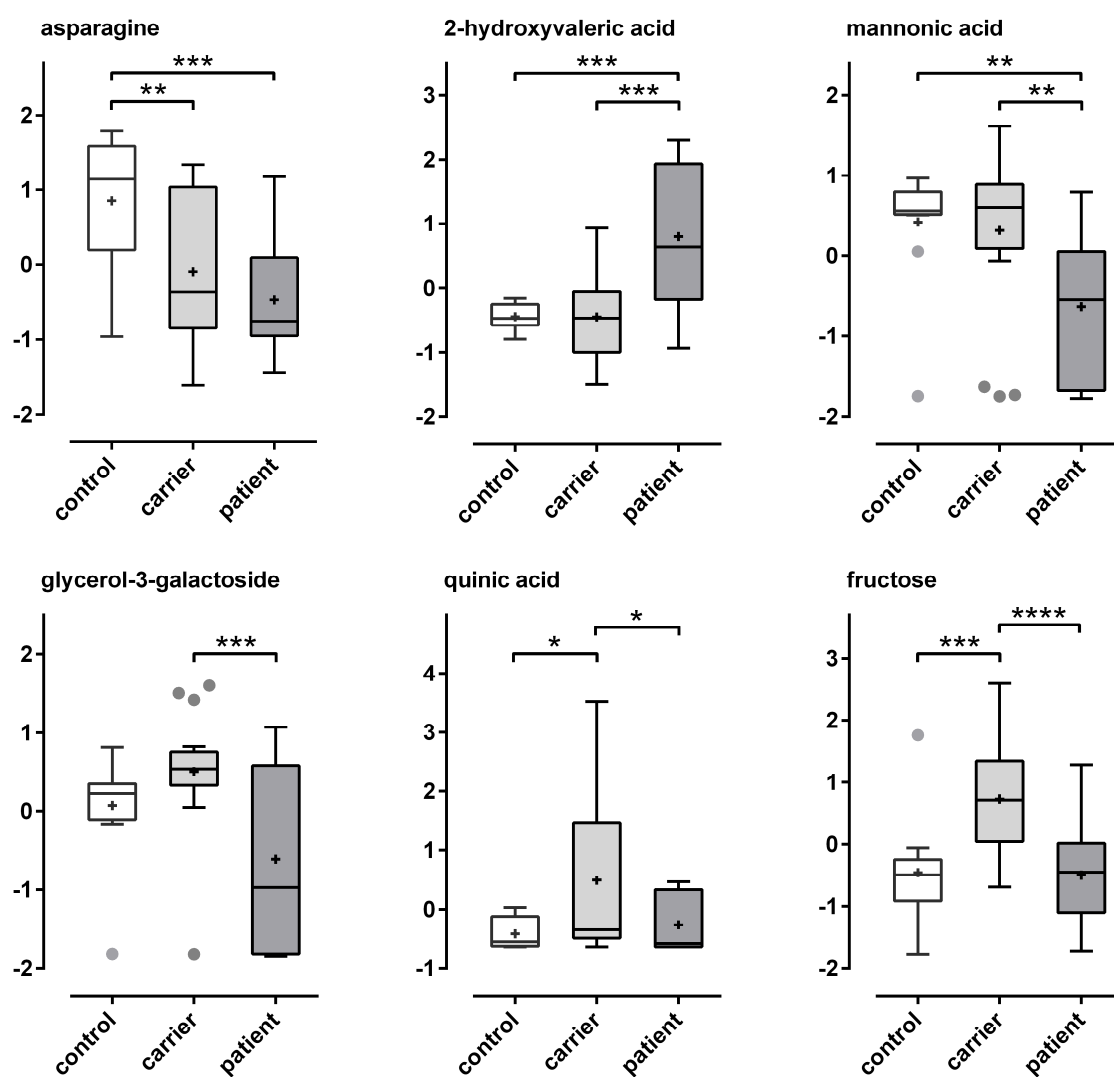

Figure S1. Boxplots of further metabolites altered PKAN erythrocyte.

Supplement: Supplementary file 1 [file biomolecules-12-00325-s001.zip › Figure S1.pdf]
